# Morphological study of the posterior osseous structures of subaxial cervical spine in a population from northeastern China

**DOI:** 10.1186/s13018-015-0194-8

**Published:** 2015-04-21

**Authors:** Zhenyu Wang, Jiali Leng, Jianhua Liu, Yi Liu

**Affiliations:** Department of Spinal Surgery, The First Hospital of Jilin University, No.71, Xinmin Avenue, Chaoyang District, Changchun, Jilin Province 130021 People’s Republic of China; Department of Radiology, The Second Hospital of Jilin University, No.218, Ziqiang Street, Changchun, Jilin Province 130041 People’s Republic of China

**Keywords:** Northeastern Chinese, Subaxial cervical spine, Lateral mass, Lamina, Spinous process, Spinal canal

## Abstract

**Background:**

Laminar screws and lateral mass screws have been increasingly used in the treatment of cervical diseases. The purpose of this study is to determine the morphological characteristics of the posterior anatomical structures of the subaxial cervical vertebrae in a northeastern Chinese population.

**Methods:**

Sixty-one consecutive patients underwent cervical spine computed tomography (CT). We analyzed a total of 610 axial images and 61 sagittal images. The following parameters were measured: lamina outer width (LOW), lamina inner width (LIW), lamina axis length (LAL), lamina transverse angle (LTA), lateral mass longitudinal diameter (LMLD), lateral mass transverse diameter (LMTD), sagittal spinous process length (SSPL), axial spinous process length (ASPL), spinal canal transverse diameter (SCTD), spinal canal longitudinal diameter (SCLD), osseous spinal canal area (OSCA), and Pavlov ratio (PR). The participants were classified into male and female groups and developmental canal stenosis (DCS; PR ≤0.75) and non-DCS (NDCS; PR >0.75) groups.

**Results:**

Significant differences were observed among the different vertebral levels for almost all evaluated parameters, except for LTA and OSCA. Statistical differences were found between the right and left sides in all parameters, except for LIW and LOW. All linear parameters, except for SCLD and the angular parameter LTA, significantly differed between the sexes. Significant differences were found between the DCS and NDCS groups in terms of all parameters, except for SCTD.

**Conclusions:**

Various measurements of the posterior structures of subaxial cervical vertebrae differed between the left and right sides, females and males, and the DCS and NDCS groups. Different techniques for lateral mass screw insertion should be used according to different vertebral level. Only C7 laminar may be able to safely accommodate a 2.5-mm translaminar screw. The study data can help doctors to make better surgical decisions and develop more appropriate implants for northeastern Chinese patients.

## Introduction

Various types of cervical spinal instrumentations such as laminar screws and lateral mass screws have been developed and have enabled more rigid fixation of the cervical spine and correction of malalignment via a posterior-only approach, especially, in the case of the subaxial cervical spine [1-6]. One of the most frequent and complex procedures involving this part of the spine is the placement of transpedicular screws [7-10]. Laminar screws and lateral mass screws have been increasingly used in the treatment of cervical diseases [2-5,11,12]. However, these techniques are beset with the risk of significant neurologic and vascular injury [13-16].

It is essential that the implants used for these operations are appropriately designed and positioned. All these implants are closely related to the morphological characteristics of posterior cervical osseous structures, which include the pedicles, laminae, and spinous processes. Knowing the dimensions of these structures is a prerequisite for the development of appropriate implants. Furthermore, ethnic variations have been reported in these dimensions [8,17-20], and to date, there have been no morphometric studies of this area in the northeastern Chinese population. Therefore, the objective of this study is to determine the morphometric characteristics of the subaxial cervical vertebrae in northeastern Chinese persons.

With rapid advances in imaging technology, thin-slice computed tomography (CT) scans and three-dimensional reconstruction techniques have enabled the detailed study of the morphology of the subaxial cervical spine [21]. This study aimed to analyze the features of the posterior structures of the subaxial cervical spine in northeastern Chinese persons by using high-resolution CT scans.

## Materials and methods

From July 2011 to July 2014, 61 patients complaining neck pain without neurological deficits, congenital deformities, trauma of the spine, and history of spinal surgery were enrolled in this study. All these patients underwent high-resolution CT scanning of the cervical spine. There were 24 women and 37 men. Their mean age was 53.2 ± 9.9 years (range, 27–71 years). Patients with congenital deformities, trauma, ossification of the posterior longitudinal ligament, ossification of the ligamentum flavum, rheumatoid arthritis, infectious spondylitis, spinal tumors, or prior spine surgery were excluded. All the patients provided informed consent. And the study was approved by the Medical Ethics Committee of our hospital.

All CT scans were obtained using a high-resolution CT device (Philips, 256-slice CT scanner, The Netherlands). The imaging data were obtained in 0.5-mm slices from the level of C1 to C7. All images were routinely reformatted into axial planes parallel to the endplates of the vertebral body. Axial images containing the largest pedicle diameter and lamina diameter were selected for the C3 to C7 vertebrae. The largest middle sagittal plane (LMSP) image of each patient was also selected. In total, we analyzed 610 axial images and 61 sagittal images. The following parameters were measured:

1. Laminae, lateral mass, and spinous process parameters: lamina outer width (LOW), which is the perpendicular distance between the medial and lateral cortical bones of the lamina isthmus; lamina inner width (LIW), which is the perpendicular distance between the medial and lateral cancellous bones of the lamina isthmus; lamina axis length (LAL); lamina transverse angle (LTA), which is the angle between the lamina axis and the midline of the vertebral body; lateral mass longitudinal diameter (LMLD), which is the distance from the posterior cortex of the lateral mass to the posterior edge of the transverse foramen; lateral mass transverse diameter (LMTD), which is the distance from the lateral cortex of the lateral mass to the medial edge of the osseous spinal canal; sagittal spinous process length (SSPL), which is the length of spinous process on sagittal plane; and axial spinous process length (ASPL), which is the length of spinous process on axial plane.
2. Spinal canal parameters: spinal canal longitudinal diameter (SCLD); spinal canal transverse diameter (SCTD); and osseous spinal canal area (OSCA).

**Figure 1:**
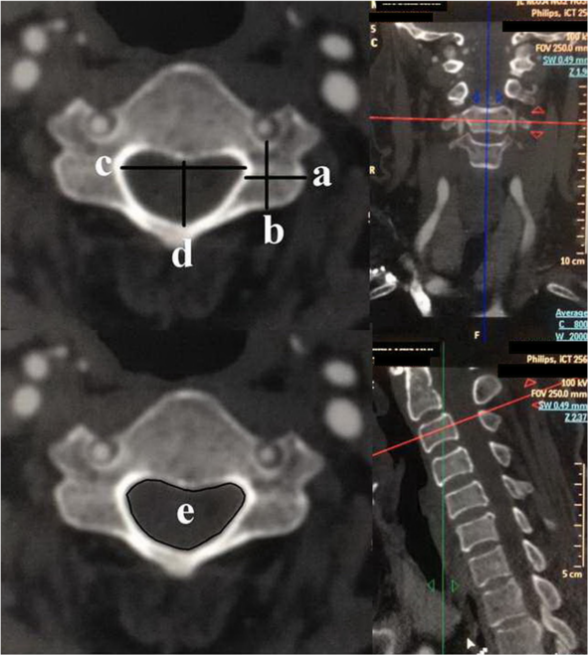
Measurements of lateral mass and spinal canal parameters.Measurement of (a) lateral mass longitudinal diameter (LMTD); (b) lateral mass longitudinal diameter (LMLD); (c) spinal canal transverse diameter (SCTD); (d) spinal canal longitudinal diameter (SCLD); and (e) osseous spinal canal area (OSCA).

**Figure 2:**
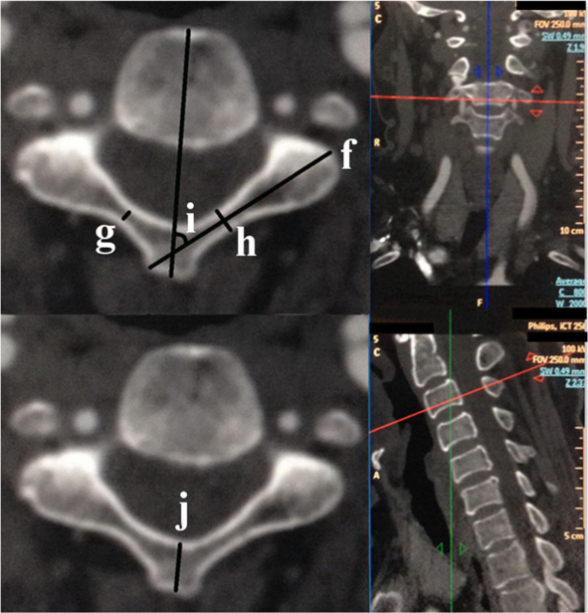
Measurements of lamina and axial spinous process parameters. Measurement of (f) lamina axis length (LAL); (g) lamina inner width (LIW); (h) lamina outer width (LOW); (i) lamina transverse angle (LTA); and (j) axial spinous process length (ASPL).

**Figure 3:**
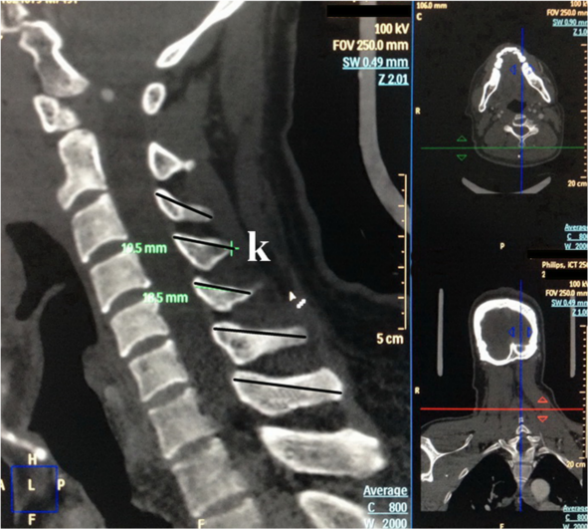
Measurement of (k) sagittal spinous process length (SSPL).

Among these parameters, LMTD, LMLD, ASPL, SCTD, SCLD, and OSCA were measured on the largest pedicle diameter plane (LPDP) parallel to the endplate of the investigated vertebra (Figure 1). LIW, LOW, LTA, and LAL were measured on the largest laminar diameter plane (LLDP) parallel to the endplate of the investigated vertebra (Figure 2). SSPL was measured on the LMSP (Figure 3). To determine whether developmental cervical spinal canal stenosis was present, we also measured the mean Pavlov ratio (PR), which is the mean of the PRs at each level from C3 to C7. Two independent observers measured each morphometric parameter in consensus, by using a digital imaging and communications in medicine (DICOM) viewer, electronic calipers, and a DICOM workstation. In our study, we investigated the reliability of the measurement techniques, and the intra- and interobserver agreement was good to excellent for each parameter (k >0.80).

The participants were classified into a female group (24 subjects) and a male group (37 subjects). They were also classified into a developmental canal stenosis (DCS) group (15 subjects, PR ≤0.75) and a non-DCS (NDCS) group (46 subjects, PR >0.75).

The Statistical Package for the Social Sciences (SPSS, version 17.0) was used. Values are represented as mean ± standard deviation. Single-factor analysis of variance was used to determine differences among the different vertebral levels, and the student *t* test was used to determine differences between different groups (female vs. male, and DCS vs. NDCS) with regard to all morphometric parameters. A significance level of 0.05 was adopted.

## Results

**Figure 4:**
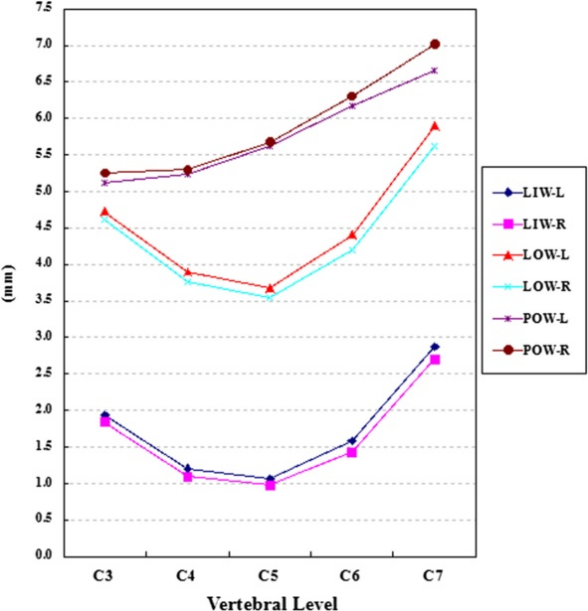
Dimensions of linear parameters, part 1. LIW: lamina inner width; LOW: lamina outer width; POW: pedicle outer width; L: left side; R: right side.

**Figure 5:**
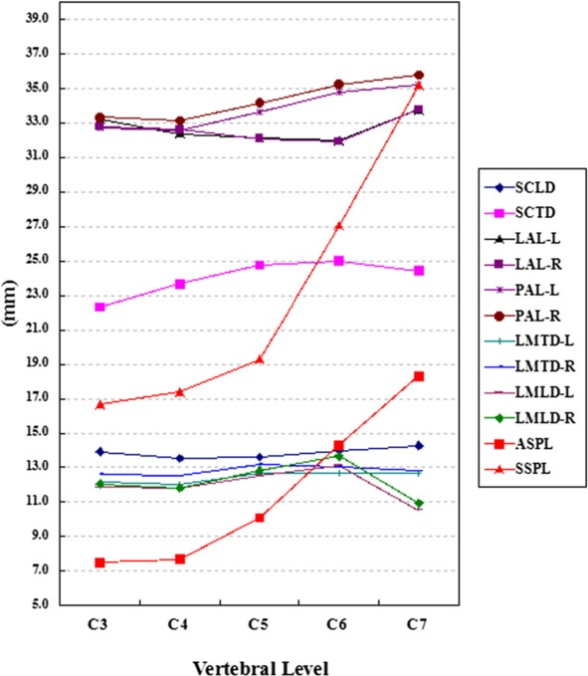
Dimensions of linear parameters, part 2. SCLD: spinal canal longitudinal diameter; SCTD: spinal canal transverse diameter; LAL: lamina axis length; PAL: pedicle axis length; LMTD: lateral mass transverse diameter; LMLD: lateral mass longitudinal diameter. ASPL: axial spinous process length; SSPL: sagittal spinous process length; L: Left side; R: right side.

**Figure 6:**
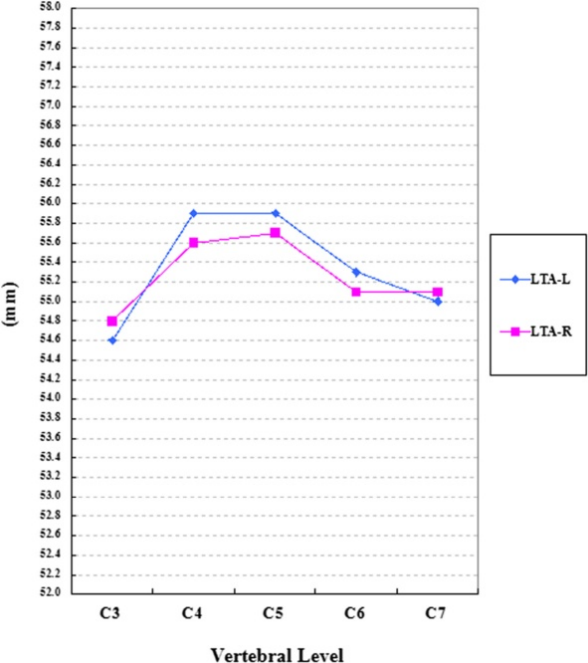
Dimensions of angular parameters. LTA: Lamina transverse angle, which is the angle between the lamina axis and the midline of the vertebral body; L: Left side; R: right side.

We studied the CT data, including the 610 axial and 61 sagittal images, from the 61 patients. The lamina, lateral mass, and spinous process parameters are presented in Table 1. The spinal canal parameters are presented in Table 2. General trends of the parameters are presented in Figures 4, 5, and 6.

**Table 1:** **Dimensions of lamina, lateral mass, and spinous process parameters at each level**

**Level**		**LIW**		**LOW**		**LAL**		**LTA**		**LMTD**		**LMLD**		**SSPL**	**ASPL**
		**Left**	**Right**	**Left**	**Right**	**Left**	**Right**	**Left**	**Right**	**Left**	**Right**	**Left**	**Right**		
C3		1.9 ± 0.9	1.8 ± 0.8	4.7 ± 1.1	4.6 ± 1.1	33.2 ± 2.9	32.8 ± 3.0	54.6 ± 2.6	54.8 ± 2.7	12.2 ± 1.4	12.6 ± 1.5	11.9 ± 1.3	12.1 ± 1.2	16.7 ± 3.3	7.5 ± 3.1
	F	1.5 ± 0.9	1.3 ± 0.8	4.2 ± 1.1	4.1 ± 1.1	31.6 ± 2.7	31.0 ± 2.7	55.0 ± 2.5	55.2 ± 2.6	11.1 ± 1.3	11.6 ± 1.2	11.1 ± 1.1	11.4 ± 1.0	14.8 ± 2.9	7.1 ± 1.7
	M	2.2 ± 0.8	2.2 ± 0.9	5.1 ± 1.0	4.9 ± 0.9	34.2 ± 2.6	34.0 ± 2.5	54.5 ± 2.7	54.5 ± 2.9	12.8 ± 1.1	13.3 ± 1.3	12.4 ± 1.2	12.5 ± 1.1	17.9 ± 3.0	8.8 ± 2.1
	DCS	2.0 ± 0.3	1.9 ± 0.4	5.0 ± 0.7	4.7 ± 0.7	33.8 ± 2.4	33.4 ± 2.0	56.2 ± 2.6	56.0 ± 2.7	12.8 ± 0.8	12.9 ± 1.1	11.9 ± 1.0	11.9 ± 0.9	17.6 ± 3.0	8.6 ± 1.4
	NDCS	1.9 ± 1.1	1.8 ± 1.1	4.6 ± 1.2	4.6 ± 1.2	33.0 ± 3.1	32.6 ± 3.2	54.3 ± 2.7	54.3 ± 2.3	12.0 ± 1.5	12.5 ± 1.6	11.8 ± 1.4	12.1 ± 1.3	16.4 ± 3.4	8.0 ± 1.9
C4		1.2 ± 0.8	1.1 ± 0.7	3.9 ± 0.9	3.8 ± 0.9	32.4 ± 2.4	32.6 ± 2.5	55.0 ± 2.3	55.1 ± 2.5	12.1 ± 1.4	12.5 ± 1.4	11.8 ± 1.2	11.8 ± 1.2	17.4 ± 3.1	7.7 ± 2.9
	F	1.0 ± 0.8	0.9 ± 0.7	3.6 ± 1.0	3.6 ± 1.0	30.9 ± 2.1	31.3 ± 2.0	55.6 ± 2.6	55.4 ± 2.4	11.0 ± 1.2	11.6 ± 1.3	11.2 ± 1.0	11.2 ± 0.9	15.7 ± 2.9	7.7 ± 2.2
	M	1.3 ± 0.7	1.2 ± 0.7	4.1 ± 0.8	3.9 ± 0.8	33.3 ± 2.2	33.5 ± 2.5	55.5 ± 2.5	55.6 ± 2.1	12.7 ± 1.1	13.2 ± 1.0	12.2 ± 1.1	12.3 ± 1.2	18.4 ± 2.8	9.4 ± 1.8
	DCS	1.3 ± 0.5	1.2 ± 0.4	4.1 ± 0.7	3.9 ± 0.7	32.6 ± 2.4	33.0 ± 2.3	55.4 ± 2.3	55.6 ± 2.5	12.2 ± 1.1	12.9 ± 0.7	12.0 ± 1.1	11.9 ± 1.3	17.2 ± 2.9	8.8 ± 1.7
	NDCS	1.2 ± 0.8	1.1 ± 0.8	3.8 ± 1.0	3.7 ± 0.9	32.3 ± 2.5	32.5 ± 2.6	55.5 ± 2.7	55.5 ± 2.6	12.0 ± 1.5	12.4 ± 1.5	11.7 ± 1.2	11.8 ± 1.2	17.4 ± 3.2	8.9 ± 1.9
C5		1.1 ± 0.7	1.0 ± 0.6	3.7 ± 0.9	3.5 ± 0.8	32.1 ± 2.5	32.1 ± 2.6	55.8 ± 3.0	55.6 ± 2.7	12.6 ± 1.5	13.2 ± 1.5	12.6 ± 1.3	12.8 ± 1.3	19.3 ± 3.3	10.1 ± 3.2
	F	0.8 ± 0.8	0.8 ± 0.7	3.3 ± 1.1	3.3 ± 1.0	30.7 ± 1.8	30.4 ± 2.1	56.0 ± 2.9	56.3 ± 2.8	11.6 ± 0.9	12.0 ± 0.7	11.9 ± 1.4	12.1 ± 1.3	17.7 ± 3.0	9.2 ± 2.1
	M	1.2 ± 0.6	1.1 ± 0.5	3.9 ± 0.8	3.7 ± 0.6	33.1 ± 2.4	33.2 ± 2.3	55.5 ± 2.6	55.6 ± 2.8	13.4 ± 1.4	14.0 ± 1.4	13.0 ± 1.0	13.3 ± 1.1	20.3 ± 3.0	10.4 ± 2.3
	DCS	1.1 ± 0.6	0.9 ± 0.5	4.0 ± 0.8	3.7 ± 0.8	31.7 ± 2.4	31.8 ± 2.4	56.9 ± 3.2	56.8 ± 3.0	12.9 ± 1.6	13.2 ± 1.4	13.2 ± 1.0	13.6 ± 0.9	18.7 ± 3.3	9.7 ± 2.0
	NDCS	1.0 ± 0.7	1.0 ± 0.7	3.6 ± 1.0	3.5 ± 0.8	32.3 ± 2.5	32.2 ± 2.7	55.6 ± 2.7	55.4 ± 2.6	12.6 ± 1.5	13.2 ± 1.6	12.4 ± 1.3	12.6 ± 1.3	19.5 ± 3.3	9.9 ± 1.9
C6		1.6 ± 0.8	1.4 ± 0.7	4.4 ± 1.0	4.2 ± 1.0	32.0 ± 2.8	31.9 ± 2.8	55.2 ± 3.3	55.3 ± 3.2	12.7 ± 1.6	13.0 ± 1.5	13.1 ± 1.4	13.7 ± 1.4	27.1 ± 4.9	14.3 ± 4.2
	F	1.2 ± 0.8	1.2 ± 0.9	3.9 ± 1.0	3.9 ± 1.2	30.1 ± 1.9	30.3 ± 2.1	56.1 ± 2.1	55.9 ± 2.3	11.5 ± 0.8	12.0 ± 0.9	12.5 ± 1.7	12.7 ± 1.3	24.9 ± 4.4	13.5 ± 2.5
	M	1.8 ± 0.7	1.6 ± 0.6	4.7 ± 0.7	4.4 ± 0.7	33.2 ± 2.7	33.0 ± 2.7	54.6 ± 3.4	54.6 ± 3.6	13.4 ± 1.5	13.7 ± 1.4	13.5 ± 0.9	14.3 ± 1.1	28.4 ± 4.9	14.1 ± 2.6
	DCS	1.5 ± 0.6	1.4 ± 0.5	4.4 ± 0.8	4.2 ± 0.9	31.2 ± 2.6	30.5 ± 2.2	56.9 ± 3.6	57.1 ± 3.4	12.5 ± 1.6	12.7 ± 1.1	14.0 ± 1.1	14.1 ± 1.0	26.2 ± 4.0	13.6 ± 2.1
	NDCS	1.6 ± 0.8	1.4 ± 0.8	4.4 ± 1.0	4.2 ± 1.0	32.2 ± 2.8	32.4 ± 2.8	54.5 ± 2.6	54.6 ± 2.7	12.7 ± 1.6	13.1 ± 1.6	12.8 ± 1.3	13.5 ± 1.5	27.3 ± 5.2	14.2 ± 2.4
C7		2.9 ± 1.1	2.7 ± 1.0	5.9 ± 1.3	5.6 ± 1.3	33.7 ± 2.4	33.8 ± 2.6	55.1 ± 2.7	54.9 ± 2.6	12.7 ± 1.3	12.8 ± 1.4	10.6 ± 1.5	10.9 ± 1.4	35.2 ± 3.4	18.3 ± 3.0
	F	2.3 ± 1.0	2.3 ± 1.2	5.1 ± 1.3	5.0 ± 1.4	32.0 ± 1.7	32.0 ± 1.9	55.5 ± 2.6	56.1 ± 2.5	11.7 ± 0.8	11.9 ± 1.2	9.9 ± 1.4	10.4 ± 1.2	33.0 ± 3.5	16.8 ± 1.9
	M	3.2 ± 0.9	3.0 ± 0.8	6.4 ± 1.1	6.0 ± 0.9	34.9 ± 2.1	34.9 ± 2.4	54.7 ± 3.0	55.2 ± 3.1	13.3 ± 1.1	13.4 ± 1.2	10.9 ± 1.4	11.3 ± 1.3	36.6 ± 2.5	19.0 ± 1.8
	DCS	2.9 ± 1.0	2.6 ± 1.0	6.0 ± 1.3	5.6 ± 1.3	33.4 ± 2.4	33.0 ± 2.8	55.8 ± 2.2	56.0 ± 2.4	12.9 ± 1.1	13.2 ± 1.1	10.5 ± 1.4	11.0 ± 1.3	35.7 ± 2.6	17.5 ± 1.7
	NDCS	2.9 ± 1.1	2.7 ± 1.0	5.9 ± 1.4	5.6 ± 1.3	33.9 ± 2.4	34.0 ± 2.5	54.7 ± 2.4	55.0 ± 2.5	12.6 ± 1.3	12.7 ± 1.5	10.6 ± 1.6	10.9 ± 1.4	35.0 ± 3.6	17.5 ± 1.9

*LIW* lamina inner width, *LOW* lamina outer width, *LAL* lamina axis length, *LTA* lamina transverse angle, *LMTD* lateral mass transverse diameter, *LMLD* lateral mass longitudinal diameter, *SSPL* sagittal spinous process length, *ASPL* axial spinous process, *L* left side, *R* right side, *F* female, *M* male, *DCS* developmental cervical stenosis, *NDCS* nondevelopmental cervical stenosis.

**Table 2:** **Dimensions of spinal canal parameters at each level**

**Level**		**SCTD**	**SCLD**	**OSCA**
C3		22.3 ± 1.3	13.9 ± 1.2	225.1 ± 28.4
	F	21.7 ± 1.0	14.0 ± 1.4	221.0 ± 32.8
	M	22.7 ± 1.2	13.9 ± 1.0	227.7 ± 25.2
	DCS	22.1 ± 0.9	13.1 ± 1.0	214.8 ± 16.9
	NDCS	22.4 ± 1.4	14.2 ± 1.1	228.4 ± 30.6
C4		23.7 ± 1.7	13.5 ± 1.3	221.8 ± 29.1
	F	22.7 ± 1.5	13.6 ± 1.5	214.9 ± 34.2
	M	24.3 ± 1.6	13.5 ± 1.2	226.2 ± 24.7
	DCS	24.2 ± 1.5	12.6 ± 1.0	210.8 ± 20.3
	NDCS	23.5 ± 1.8	13.8 ± 1.3	225.3 ± 30.7
C5		24.8 ± 1.8	13.6 ± 1.3	229.1 ± 30.9
	F	23.9 ± 1.7	13.6 ± 1.4	219.8 ± 29.4
	M	25.4 ± 1.6	13.6 ± 1.3	235.2 ± 30.7
	DCS	25.0 ± 1.7	12.6 ± 1.3	211.9 ± 23.6
	NDCS	24.7 ± 1.9	13.9 ± 1.2	234.8 ± 31.2
C6		25.0 ± 1.8	14.0 ± 1.2	237.0 ± 31.4
	F	24.1 ± 1.5	13.7 ± 1.2	224.9 ± 30.1
	M	25.6 ± 1.7	14.2 ± 1.1	244.9 ± 30.1
	DCS	25.4 ± 1.7	13.1 ± 1.0	222.1 ± 25.1
	NDCS	24.8 ± 1.9	14.3 ± 1.1	241.9 ± 32.0
C7		24.4 ± 1.8	14.3 ± 1.3	231.1 ± 28.7
	F	23.7 ± 1.5	13.8 ± 1.0	218.2 ± 24.3
	M	24.9 ± 1.9	14.6 ± 1.3	239.4 ± 28.5
	DCS	24.8 ± 1.9	13.2 ± 1.2	219.5 ± 27.6
	NDCS	24.3 ± 1.8	14.6 ± 1.1	234.9 ± 28.3

*SCTD* spinal canal transverse diameter, *SCLD* spinal canal longitudinal diameter, *OSCA* osseous spinal canal area, *L* left side, *R* right side, *F* female, *M* male, *DCS* developmental cervical stenosis, *NDCS* nondevelopmental cervical stenosis.

Significant differences were observed among the different vertebral levels for almost all the evaluated parameters (*P* <0.05), except for LTA and OSCA. Statistical differences were found in all levels between the right and left sides for most evaluated parameters (*P* <0.05), except for LIW and LOW. All linear parameters (LOW, LIW, LAL, SCTD, SCLD, SSPL, and ASPL), but not the angular parameters (LTA) differed significantly between the sexes (*P* <0.05). Almost all parameters showed significant differences between the DCS group and the NDCS group at different levels, except for SCTD. And SCLD showed significant differences, except at the C3 and C4 levels. OSCA showed significant differences only at the C5 and C6 levels. LMLD and LMTD showed significant differences at the C5, C6 and C7 level, respectively.

### Lamina, lateral mass, and spinous process parameters

Table 1 shows the results of LIW, LOW, LAL, LTA, LMTD, LMLD, SSPL, and ASPL. The general trends of these parameters are shown in Figures 4 and 5. Significant differences were found between the two sides for LMTD at all levels, except C7, and for LMLD at all levels, except C4. C6 and C5 had the largest measurements of LMLD (*P* <0.05), while the smallest measurements were observed at C7 level (*P* <0.05). C5, C6, and C7 had longer LMTD (*P* <0.05). Significant differences between LMTD and LMLD were observed at the C4 level (only on left side) and C5 level (on both left and right sides).

As a general trend, the mean LIW and LOW decreased from C3 to C5 and increased from C5 to C7 on both sides. The smallest LIW and LOW were both observed at the C5 level (*P* <0.05). The largest LIW and LOW were measured at the C7 level (*P* <0.05). Both the mean SSPL and ASPL increased steadily from C3 to C7.

### Spinal canal parameters

Table 2 shows the results of SCTD, SCLD, and OSCA. Figure 5 shows the general trends of SCTD and SCLD. As a general trend, the mean SCTD increased from C3 to C6 and decreased from C6 to C7. The largest SCTD was measured at the C6 level (*P* <0.05). The mean SCLD decreased from C3 to C4 and increased from C4 to C7. The largest SCLD was measured at the C7 level (*P* <0.05). The mean OSCA fluctuated from C3 to C7, and no significant differences were found among the different vertebral levels (*P* >0.05).

## Discussion

Several methods of fixation have been used in the subaxial cervical spine, including pedicle screws, lateral mass screws, and laminar screws. Therefore, detailed anatomical data of the subaxial cervical spine for the accurate implantation is urgently required. Anatomical measurements of the critical morphometric characteristics of the subaxial cervical spine related to a variety of fixation techniques have been studied by analyzing data from direct measurement of cadavers, CT scans, and 3D reconstructions [10]. Furthermore, differences in vertebral dimensions have been shown to exist among different races [10,22]. We therefore analyzed the anatomical dimensions of the subaxial cervical spine in the northeastern Chinese population.

Most anatomical studies on the spinal column seem to be based on the unverified assumption that there are no significant differences in anatomical parameters between the right and left sides. Therefore, the results of most anatomical studies of the spinal column incorrectly report only a single measurement rather than two different data points for both sides. In our study, only two anatomical parameters, i.e., LIW and LOW, showed no significant differences between the two sides. This result may indicate that spinal surgeons should consider the difference between the left and right sides to facilitate the safe placement of various types of spinal implants rather than always pursuing symmetrical manipulation.

Gender differences between linear cervical laminar parameters have been reported [12]. However, detailed gender-based differences in the cervical pedicle, spinal canal, and angular cervical laminar parameters are rarely reported. We found that male subjects had significantly larger diameters than female subjects for all linear parameters, except for SCLD, and the angular parameter. This suggests that different size implants should be selected, but a similar insertion angle should be applied during cervical fixation surgery in patients of different sexes.

In this study, we determined the specific anatomical dimensions of the adult subaxial cervical laminae, lateral masses, spinous process, and spinal canal parameters using CT radiographic analysis.

Lateral mass screws are increasingly commonly used for posterior fixation of the cervical spine to treat traumatic and degenerative conditions [23]. The numerous surgical techniques can be divided into where the trajectories are perpendicular to the posterior lateral mass surface (i.e., the Roy-Camille technique [24,25]) and those where the screw trajectory is rather parallel to the facet joint and more lateral on the axial plane (e.g., Magerl [26], Anderson [27], An [28], and Cheng [29]) [30]. It is widely accepted that the bio-mechanical properties differ between the Roy-Camille and Magerl techniques. Screw pullout strength and sagittal stiffness are significantly greater with the Magerl techniques [31,32]. An observation was attributed to a superior bony anchorage due to longer lateral mass screw trajectories [30]. According to our study, C6 and C5 had the largest measurements of LMLD (*P* <0.05), while the smallest measurements were observed at C7 level. Therefore, both the Roy-Camille and Magerl techniques might be able to be used at C6 and C5 level. However, the Magerl technique was recommended at C7 level due to the possible shortest lateral mass screw trajectories. Moreover, C5, C6, and C7 had longer LMTD (*P* <0.05) which meant more oblique lateral mass trajectory applied in the Magerl and Cheng techniques might be accommodated.

Most current studies on laminar anatomy in the adult population have focused on the LOW [12,21] rather than the LIW. Limited investigation has been performed within the adult population to understand the core data of laminar anatomy: clinical surgeons consider that the LIW is more important for safe laminar screw placement. This is the main concern of our study. We found that only the C7 lamina, but not the C3–C6 laminae, may be able to safely accommodate a 2.5-mm translaminar screw. Our suggestion is different to that of Alvin et al. [12] and of Ji et al. [33]. In our opinion this difference is due to different focus of study. Our focus was the LIW rather than the LOW or the thickness of lamina. Furthermore, according to our study, the smallest LIW and LOW were both observed at the C5 level. Therefore, translaminar implants should be more carefully inserted at the C5 level. In other words, preoperative CT scans of all cervical vertebral levels are indispensable for translaminar screw insertion.

Differences between the dimensions of the DCS and NDCS groups show evident variation among different cervical vertebral levels. No significant regularity was found. Our results differ from those of Miyazaki et al. [21].

We found that usually, the axial plane of the largest pedicle diameter did not present the largest laminar diameter and vice versa. Therefore, we did not apply the method, which has been used in many studies, in which measurements are obtained from only one axial plane [12,21,34]. Instead, we measured dimensions from two axial planes.

Some of the results of our study were compared with those of the earlier studies of Panjabi MM et al. [35], Tan SH et al. [17], Stemper BD et al. [36], Abdullah KG et al. [34], Alvin MD et al. [12], and Yusof MI et al. [37] (Table 3 and Table 4). In general, the results presented in our study agree well with those of the earlier studies, but there were some marked differences. The LMLD dimensions from our study were significantly larger than those of Abdullah KG et al. because our data were measured from the posterior cortex of the lateral mass to the posterior edge of the transverse foramen instead of the distance from the dorsal to ventral cortices through the center of the lateral mass.

**Table 3:** **Comparison of lamina and lateral mass parameters**

**Parameter**	**Level**	**Gender**	**Our study**	**Alvin (2012)** **[** 12 **]**	**Stemper (2008)** **[** 36 **]**	**Abdullah (2009)** **[** 34 **]**	**Yusof (2012)** **[** 37 **]**
LIW	C3		1.9	—	—	—	2.0
	C4		1.1	—	—	—	1.7
	C5		1.0	—	—	—	1.9
	C6		1.0	—	—	—	2.3
	C7		2.8	—	—	—	3.4
LOW	C3	F	4.2	4.1	—	—	3.5
		M	5.0	4.2	—	—	
	C4	F	3.6	3.6	—	—	3.1
		M	4.0	3.8	—	—	
	C5	F	3.3	3.3	—	—	3.1
		M	3.8	3.6	—	—	
	C6	F	3.9	3.9	—	—	3.9
		M	4.6	4.3	—	—	
	C7	F	5.1	5.9	—	—	5.8
		M	6.2	6.3	—	—	
LAL	C3		33.0	—	—	—	31.2
	C4		32.5	—	—	—	31.5
	C5		32.1	—	—	—	32.1
	C6		31.9	—	—	—	30.6
	C7		33.8	—	—	—	32.2
LTA	C3		54.7	—	—	—	50.8
	C4		55.5	—	—	—	51.4
	C5		55.8	—	—	—	50.9
	C6		55.2	—	—	—	51.1
	C7		55.0	—	—	—	50.5
LMTD	C3	F	11.4	—	10.0	—	—
		M	13.1	—	11.1	—	—
	C4	F	11.3	—	10.3	—	—
		M	13.0	—	11.4	—	—
	C5	F	11.8	—	11.0	11.0	—
		M	13.7	—	12.4	12.9	—
	C6	F	11.8	—	11.1	11.4	—
		M	13.6	—	12.8	12.8	—
	C7	F	11.8	—	10.3	10.5	—
		M	13.4	—	11.8	11.5	—
LMLD	C3	F	11.3	—	—	—	—
		M	12.5	—	—	—	—
	C4	F	11.2	—	—	—	—
		M	12.3	—	—	—	—
	C5	F	12.0	—	—	8.8	—
		M	13.2	—	—	9.2	—
	C6	F	12.6	—	—	8.6	—
		M	13.9	—	—	10.5	—
	C7	F	10.2	—	—	9.6	—
		M	11.1	—	—	10.8	—

LIW indicates lamina inner width; LOW, lamina outer width; LAL, lamina axis length; LTA, lamina transverse angle; LMTD, lateral mass transverse diameter; LMLD, lateral mass longitudinal diameter; F, female; M, male.

**Table 4:** **Comparison of spinal process and spinal canal parameters**

**Parameter**	**Level**	**Our study**	**Panjabi (1991) [** 35 **]**	**Tan (2004) [** 17 **]**
SSPL	C3	16.7	29.6	25.6
	C4	17.4	30.3	30.3
	C5	19.3	28.5	33.6
	C6	27.1	34.2	40.5
	C7	35.2	45.7	46.9
SCTD	C3	22.3	22.9	19.2
	C4	23.7	24.7	19.3
	C5	24.8	24.9	20.3
	C6	25.0	25.8	20.6
	C7	24.4	24.5	19.7
SCLD	C3	13.9	16.2	10.3
	C4	13.5	17.7	10.3
	C5	13.6	17.4	10.3
	C6	14.0	18.1	10.3
	C7	14.3	15.2	11.0
OSCA	C3	225.1	248.7	149.7
	C4	221.8	272.0	159.9
	C5	229.1	249.5	166.8
	C6	237.0	266.5	163.7
	C7	231.1	223.8	167.5

*SSPL* sagittal spinous process length, *SCTD* spinal canal transverse diameter, *SCLD* spinal canal longitudinal diameter, *OSCA* osseous spinal canal area.

The spinal canal areas of our study differed significantly from those of Tan et al. [17] but similar to those of Panjabi et al. [35].

The SSPL presented herein were significantly less than those observed by Panjabi [35] et al. and Tan et al. [17] by about 10 mm. In our opinion, this apparent discrepancy is due to the difference in where the length was measured. In our study, the SSPL was measured on the largest middle sagittal plane which meant the tips of spinous process could not be measured due to its special bifid or slightly bent shapes.

Our study population was comprised of 61 patients all come from northeastern China and may not have been sufficiently large to be generalized to the greater population. Therefore, our study results may be applicable only to those northeastern Chinese population. Larger population coming from all parts of China even Asia enrolled in this kind of study may provide more persuasive and typical morphological results. And our center is currently preparing for launching a plan of multicenter morphological study on subaxial cervical spine of the Asian population.

We hope our results will help to improve the quality of mathematical models of the subaxial cervical spine. In addition, these data may also be used clinically, for example, in the design of surgical implants for this region of the cervical spine.

## Conclusion

The measurements of most symmetrical and bilateral anatomical structures of the subaxial cervical vertebrae differ between the left and right sides. Specific consideration should be given to these differences prior to surgical manipulation and spinal implant insertion. Different techniques for lateral mass screw insertion should be used according to different vertebral level. Only C7 laminar may be able to safely accommodate a 2.5-mm translaminar screw. The data of this study can be used to help make better surgical decisions and develop more appropriate cervical devices for the northeastern Chinese population.

### Keypoints

1. Most of the morphometric data of the subaxial cervical spine differ between the left and right sides, a finding that has not been previously reported.
2. Different implant sizes should be selected, but a similar insertion angle should be applied during cervical fixation surgery in patients of different sexes.
3. Different techniques of lateral mass screw insertion may be used in different cervical level.
4. The C7 lamina may be the only segment that can safely accommodate a translaminar screw.
5. Evident variation among different cervical vertebral levels was found between patients with and without developmental cervical stenosis. However, no significant regularity was found.
6. We recommend that spine surgeons should measure those anatomic parameter of subaxial cervical spine on 3D-CT scans for determination of safe screw size at each level before surgery.

## Abbreviations

CT: Computed tomography
PR: Pavlov ratio
DCS: Developmental canal stenosis
NDCS: Nondevelopmental canal stenosis
LMTD: Lateral mass transverse diameter
LMLD: Lateral mass longitudinal diameter
SCTD: Spinal canal transverse diameter
SCLD: Spinal canal longitudinal diameter
OSCA: Osseous spinal canal area
LIW: Lamina inner width
LOW: Lamina outer width
LAL: Lamina axis length
LTA: Lamina transverse angle
ASPL: Axial spinous process length
SSPL: Sagittal spinous process length
